# Multimedia Formats for Patient Education and Health Communication: Does User Preference Matter?

**DOI:** 10.2196/jmir.5.3.e19

**Published:** 2003-08-29

**Authors:** David Wiljer, Pam Catton

**Affiliations:** ^1^Princess Margaret HospitalUniversity Health NetworkToronto ONCanada

## Introduction

Since technology has given us new methods of delivering education to patients and health care providers, the availability of resources, formats and approaches has increased dramatically. The variety of choices is certainly reflected in the scope of Bader and Stein's valuable study comparing 5 different formats for delivering patient information [1]: a text paperback booklet, paperback booklet formatted in HTML on the Web, spoken audio files, audio files synchronized with a text Web page, and Flash multimedia (animation, spoken audio, and text).

Selecting the right resources and making the most of limited educational budgets is becoming more and more challenging. Education is also playing an increasingly-important role in cancer care since patients and their families are faced with many difficult decisions that can potentially have an enormous impact on their health and quality of life [2]. Placing patients at the center of their own care is a challenging endeavor in a system fundamentally perceived and conceptualized from the clinician-centered vantage point [3- 5]. This change requires a profound shift in the way the day-to-day business of health care is performed. The cornerstone for this change is the commitment to place patients at the center of their care, by supporting, by educating, and by empowering patients to become partners in their care.

The quantity and quality of available evidence about the efficacy of many resources and programs for patient education in cancer care is severely limited. Having to decide about providing and developing educational resources raises difficult questions for providers, educators, and administrators in health care organizations: What kind of resources should be provided? What resources will result in the best outcomes? What are the key outcomes we should be measuring? In the face of this myriad of questions, we need more data and evidence to make better and more-timely decisions [6]. Bader and Stein have made a significant contribution to the slowly-growing body of available evidence. However, the body of evidence is still inconclusive and at times contradictory. The fear of making costly errors that can impact patient care looms large.

In Bader and Stein's study, it is interesting — but not surprising — to learn that users prefer a multimedia presentation of the content. Because of the costs and resources involved in implementing multimedia and other types of software, the finding that the media itself did not have an impact on "learning" ignores the question of whether preference is a worthwhile basis for investing valuable, finite, and limited resources. Within the framework of this particular study, one might be tempted to say that the investment in multimedia does not provide a sufficient return. However, within a broader framework, the investment in multimedia programs begins to make sense from a variety of perspectives.

## Efficacy of Multimedia for Learning

Before we consider the broader perspective, note that the results of Bader and Stein's study about the efficacy of multimedia are not what one would have expected based on existing data. Within the framework of the study, Bader and Stein investigate whether a particular piece of information presented in different formats has a measurable outcome on learning and understanding, and conclude that learning occurred equally in all formats. The authors explored several potential reasons for these conclusions, including the possibility that: format does not affect learning, the technology was not optimized, the sample size was not large enough, or the pre-test and post-test instrument was not effective.

There is a growing body of evidence that demonstrates the beneficial effects of multimedia on learning [7]. In certain circumstances, cognitive theorists and researchers have demonstrated improved learning outcomes with the use of multimedia tools. Richard E. Mayer has created several learning experiments and has shown "that multimedia works — that is, at least in the case of scientific explanations, adding illustrations to text or adding animation to narration can help students to better understand the presented explanation" [7]. Bader and Stein investigated resources that met Mayer's criteria and, therefore, according to Mayer's findings should have demonstrated some positive benefits. The issue of the efficacy of different types of multimedia to enhance learning requires further research studies that will examine the merits and possible benefits of educational multimedia resources.

### Patient Preference and Patient-Centered Care

Even if learning does remain the same in all the formats of information, how do we evaluate the importance of patient preference within our decision-making framework? Does the preference for the multimedia format suggest other outcome measures that we should consider? Within a patient-centered model of care, patient preference is a core value [3,5]. Supporting a patient-centered model does not imply that all patients must prefer it or that all pertinent information should be given to patients. Rather, the system should be prepared to respond in a holistic fashion to the needs and requests of individual patients and their families; multimedia can support this.

Patient satisfaction is enormously important in most hospital organizations and creating educational resources that can contribute significantly to patient satisfaction has obvious benefits that can go a long way towards justifying the initial investment. However, we would argue that even patient satisfaction is too narrow to be used as a measure to determine the relative value of educational multimedia resources compared with more-traditional methods. The relative value of individual preferences in the context of emerging patient-centered care models must be carefully considered. Given the potential of multimedia to play a large role in many aspects of patient education and care, we believe a much-wider net is required to begin to capture the value and importance of a comprehensive multimedia program.

## Evaluating the Patient Experience

In developing resources for patients and providers, the challenges of the health care system require that we do far more than provide information. Understanding is a valuable outcome to measure, but we must consider other potential outcomes and their merits. In looking for more outcomes and measurement tools, we can draw on several models for patient-centered care for evaluating the patient experience. The Picker Institute, for example, argues that given the holistic nature of patient-centered care, patient satisfaction is not a sufficiently-broad outcome measurement; the institute has developed a patient-experience framework for measuring outcomes based on the 8 dimensions of care [8].

Based on the Picker model for measuring patient experience, we could perhaps evaluate multimedia resources based on a broader approach by considering issues such as: the quality of information and efficacy of the educational content; access to information and resources; respect for patient's values; linguistic needs and learning preferences; integration with other educational services; comfort and ease of use; and levels of emotional support (including alleviation of fear and anxiety). There is a growing body of evidence that suggests involvement in decision making leads to increased patient participation in health care [9]. Because of this, we are interested in the role that effective and well-designed multimedia resources can play in encouraging a greater participation by patients in decision making.

### Merits of Multimedia

One advantage of well-designed multimedia is flexibility. Within the context of patient-centered cancer education, the flexibility of multimedia to meet diverse challenges begins to show its real potential. Although Bader and Stein refer to the advantage of multimedia for users with different learning styles, this is only part of the total equation. Multimedia can also assist educators in overcoming linguistic, cultural, and physical barriers; in addressing different learning levels; in providing the unique experiences of patients and heath care professionals; in presenting materials in different formats and from different perspectives; in providing feedback and decision-making resources; and in tailoring and customizing information to the needs of individual patients and providers [10,11]. It is only within the broader educational, cognitive, cultural, clinical, social, ethical, financial, and personal landscape that the context for user preference emerges and the value of multimedia can truly be evaluated.

Our experience with developing a multimedia program — the Oncology Interactive Education Series (OIES) — at Princess Margaret Hospital taught us that these kinds of tools have the potential to impact many aspects of education and care. Our program covers education across the continuum of care, including: information on managing side effects, detailed information on how to do self-examinations and certain kinds of exercises, and avenues for patients to learn from other patient experiences through patient testimony.

Patients have strongly endorsed the Oncology Interactive Education Series [12] and in a survey of 105 patients, 80% stated that they would use it again, and many of them prefer it to other resources [13]. This series puts a great value on communicating not only with words, but also with images. Perhaps more importantly as far as preference is concerned, patients can access key types of information in any number of ways. Users can find basic information, view an animation, explore key elements interactively, or explore the content developed for health care professionals. They can also explore beyond the resource and find more in-depth information on vetted resources on the Internet. It has been our experience that as part of a patient-centered program, multimedia can go a long way towards supporting patients and in improving their overall experience.

## Future Directions

As we continue to gather data from studies like that of Bader and Stein and to develop more-comprehensive approaches to measuring outcomes, we can further reveal the meaning and importance of preference when developing multimedia resources and delivering patient-centered care services. The importance of multimedia for cancer education needs to be examined more thoroughly. It is no longer sufficient to investigate the efficacy of computer-based tools. We must now look carefully at the quality of the software and investigate a set of evaluative criteria that helps us understand the options for and the benefits of developing new resources. As Bader and Stein point out, Mayer and other researchers have clearly demonstrated that not all multimedia are created equal. When evaluating multimedia, we must be careful to move beyond the question of whether it is useful. The question of what makes a useful multimedia program, although much more difficult to determine, will ultimately move our discussion forward and will help us immensely to make decisions about what kinds of resources to develop and how best to implement those resources.
